# Application of starch-degradation bacteria in cigar tobacco leaf fermentation: effects on starch degradation, microbial communities and metabolic pathways

**DOI:** 10.3389/fmicb.2025.1632731

**Published:** 2025-07-21

**Authors:** Yuming Yin, Xueru Song, Yonghe Cui, Jilai Zhang, Kejian Fu, Qi Zhou, Yuxing Feng, Jiakui Huang, Chenglei Hu, Yishu Deng, Youbo Su

**Affiliations:** ^1^College of Resources and Environment, Yunnan Agricultural University, Kunming, China; ^2^Yunnan Tobacco Company, Yuxi City Corporation, Yuxi, China; ^3^Research and Development Center, Yunnan Yuntianhua Co., Ltd., Kunming, China; ^4^College of Agronomy and Biotechnology, Yunnan Agricultural University, Kunming, China; ^5^College of Architectural Engineering, Yunnan Agricultural University, Kunming, China

**Keywords:** cigar, fermentation, starch-degradation bacteria, microbial community, metabolic pathway

## Abstract

**Objective:**

This study aims to explore the effects of inoculating starch-degrading strains on the starch content and microbial metabolic pathways of cigar tobacco leaves.

**Method:**

By isolating and screening starch-degradation bacteria from the surface of Dominican tobacco leaves at the end of fermentation and applying them to the fermentation process of “*Yunxue NO. 39*” cigar tobacco leaves. The study systematically analyzed the starch content, microbial diversity and community structure, starch metabolic enzyme profiles and key metabolic pathway changes in tobacco leaves fermented for 18 and 35 days, integrating physicochemical composition, non-targeted metabolomics and metagenomics.

**Result:**

The results indicated that both strains *Bacillus pumilus* and *Bacillus velezensis* (DX and BLS) exhibited strong starch degradation capabilities. Inoculation with starch-degradation bacteria significantly enhanced the diversity of the microbial community, enriched functional microbial community (such as *Bacillus*, *Acinetobacter*, *Staphylococcus* and *Aspergillus*), markedly influenced the composition of tobacco leaf metabolites and optimized the micro-environment of tobacco leaf fermentation. Metagenomic analysis revealed that the dynamic changes of starch metabolic enzymes (such as α-amylase, β-amylase and glucoamylase) during the fermentation process were closely related to the succession of the microbial community, with *Bacillus* and *Aspergillus* promoting starch degradation through synergistic interactions. KEGG pathway analysis revealed that starch metabolism is primarily accomplished through four core pathways: starch and sucrose metabolism, amino sugar and nucleotide sugar metabolism, glycolysis/gluconeogenesis and mycolic acid biosynthesis. This study provides a theoretical basis for the quality improvement of cigar tobacco leaves and confirms the potential application value of starch-degradation bacteria in tobacco fermentation.

## 1 Introduction

Tobacco is an important economic crop. China is the world’s largest producer and consumer of tobacco, with its annual tobacco consumption accounting for approximately one-third of the global total (Fu et al., 2024; Liu et al., 2015). Cigars are a purely handcrafted tobacco product, renowned for their rich smoke, mellow and full aroma, a flavor profile that is fragrant, bitter, yet sweet (Ren et al., 2024). Currently, there is an increasing market demand for improving tobacco quality. However, there remains a significant gap between the quality of Chinese tobacco and internationally recognized high-quality tobacco. The primary factor contributing to this quality disparity is the retention of large amounts of macromolecular substances such as starch, cellulose and protein, which severely impair the overall quality of the tobacco (Banožić et al., 2020). A high starch content in tobacco leaves leads to a rigid texture, lack of smoothness and softness, reduced elasticity of cigarettes and the production of an excessively burnt odor during smoking (Zong et al., 2020), generating rough smoke (Zhong et al., 2022). Harmful substances such as acetaldehyde and acrolein are generated when starch is incompletely burned which affecting the sensory quality and safety of the product (Gong et al., 2023). Additionally, insufficient starch degradation reduces the content of aroma precursor compounds such as reducing sugars and amino acids, negatively impacting the aroma and taste of tobacco leaves (Zhang Y. et al., 2024). Therefore, reducing the starch content of tobacco leaves is of great significance for improving the quality of tobacco leaves and enhancing their usability (Chen et al., 2019).

Cigars and ordinary cigarettes differ fundamentally in their manufacturing processes. Ordinary cigarettes are made from flue-cured tobacco leaves, while cigars are a type of cigarette product made from cigar tobacco leaves that have been air curved and fermented. Renowned for their rich cultural heritage and incredible taste (Allem et al., 2019), the production process of cigars is more intricate and complex, involving key stages such as cultivation, air curving, fermentation, packaging and storage. Among these, fermentation is the core process that determines the quality of cigars (Su et al., 2024). This unique fermentation process is essentially a combination of physical, chemical and biological changes (Wu Q. et al., 2023). The abundant microbial communities on the surface of tobacco leaves, such as *Pseudomonas* and *Bacillus*, secrete enzymes to break down macromolecules like starch and proteins into smaller molecules such as monosaccharides and amino acids (Zhou et al., 2020). These degradation products are further transformed into volatile aromatic substances like 3-hydroxy-damascone and ethyl palmitate (Gong et al., 2023; Zhang M. et al., 2024), while promoting the degradation of carotenoids and the Maillard reaction, thereby forming the distinctive aroma characteristics of cigars (Si et al., 2023). Research indicates that treatment with specific bacterial strains such as *Paenibacillus amylolyticus* can significantly reduce the starch and pectin content in tobacco leaves, with a degradation rate exceeding 40%, while simultaneously increasing the content of soluble sugars and aroma compounds (Gong et al., 2023). The fermentation process also effectively degrades irritants such as nicotine and ammonia, improves the physical properties of tobacco leaves making them softer and easier to roll and reduces the content of harmful substances such as nitrates (Zhang L.-Y. et al., 2024). It is precisely through these complex microbial metabolic activities that the fermentation process plays an irreplaceable role in enhancing the aroma quality of cigars (Yao et al., 2024), improving the smoking experience and enhancing product safety (Zuo et al., 2019). This also makes microbial regulation technology a key research direction for optimizing cigar quality (Hu H. et al., 2022; Song et al., 2024).

This study successfully isolated two strains with starch-degradation capabilities from the surface of Dominican tobacco leaves post-fermentation. Through 16S rDNA amplification and phylogenetic analysis, the strains were identified as *Bacillus pumilus* and *Bacillus velezensis*. Enzyme production characteristics of these strains were determined, revealing that both *B. pumilus* and *B. velezensis* could produce amylase, with *B. velezensis* also capable of producing cellulase. These strains were then applied to the fermentation process of cigar tobacco leaves. Post-fermentation analyses of the tobacco leaves included starch content, non-targeted metabolomics and metagenomics, alongside the use of correlation network diagrams to analyze the relationships between tobacco leaf starch content, intermediate compounds of starch metabolism and microbial communities. This provides a scientific basis for clarifying the contribution of starch-degradation bacteria to the degradation of starch content in cigar tobacco leaves and for elucidating microbial metabolic pathways.

## 2 Materials and methods

### 2.1 Screening and identification of starch-degradation bacteria

In this study, the dilution spread plate method was employed to screen for starch-degradation bacteria. Sterile centrifuge tubes were used to collect samples of fermented Dominican tobacco leaves. Under aseptic conditions, 5 g of the sample was added to a conical flask containing 150 mL of sterile water, followed by constant temperature oscillation at 120 r/min for 30 min for dispersion. The supernatant was then gradiently diluted to 1 × 10^−6^, 1 × 10^−7^ and 1 × 10^−8^ and 100 μL of each dilution was spread onto Nutrient Agar (NA) medium. The plates were incubated at a constant temperature of 28°C for 3 days. Colonies with different morphologies were collected and purified on NA medium. The purified strains were inoculated onto starch hydrolysis selective medium and sodium carboxymethyl cellulose medium. After 48 h, the formation of clear zones was observed and the D/d values were measured. The larger the D/d value, the stronger the starch and cellulose degradation capabilities of the strain. D represents the diameter of the clear zone (including the colony) and d represents the diameter of the colony, the unit is millimeters. Finally, the two strains with the largest clear zones were selected as the target strains.

The DNA of the target starch-degradation bacteria was extracted using a bacterial genomic DNA extraction kit. The 16S rDNA gene of the target strain was amplified by PCR with universal primers 27F-1492 R, where the forward primer was 27F (sequence: TACGGYTACCTTGTTACGACTT) and the reverse primer was 1,492 R (sequence: AGAGTTTGATCMTGGCTCAG). The amplification length was 1.5 Kb. The amplified PCR products were sequenced by Beijing Qingke Biotechnology Co., Ltd. Input the sequence results into the NCBI (National Center for Biotechnology Information) website^1^ and use the BLAST program for nucleic acid sequence comparison. Construct the phylogenetic tree using the neighbor-joining method with MEGA11 software.

### 2.2 Experimental design for cigar fermentation

To evaluate the degradation effect of starch-degradation bacteria on the starch content of cigar tobacco leaves, two strains of starch-degradation bacteria with strong amylase production capabilities were selected and inoculated into three 200 mL bottles of Nutrient Broth (NB) culture medium, respectively. The cultures were shaken at 180 r/min for 48 h at 28°C, then centrifuged at 4,000 rpm for 20 min. The resulting bacterial cells from each strain were dissolved in 450 mL of glucose water to prepare the bacterial fermentation broth.

The experiment selected uniformly sized, mechanically undamaged and mold-free “*Yunxue NO. 39*” cigar tobacco leaves for fermentation. The experiment was divided into four treatments, with 5 kg of tobacco leaves for each treatment. The experimental treatments included the CK sprayed with 300 mL of fermentation aid (provided by Yuxi City Company of Yunnan Tobacco Company) and 300 mL of glucose water, the DX sprayed with 300 mL of fermentation aid and 300 mL of *B. pumilus* bacterial solution, the BLS sprayed with 300 mL of fermentation aid and 300 mL of *B. velezensis* bacterial solution and the DX_BLS sprayed with 300 mL of fermentation aid, 150 mL of *B. pumilus* bacterial solution and 150 mL of *B. velezensis* bacterial solution, with three replicates for each treatment. The fermentation aid and bacterial solutions were evenly sprayed on the surface of the tobacco leaves, which were then stacked, wrapped in sterile gauze and placed in a fermentation chamber at 35°C and 75% relative humidity for 35 days of fermentation. This experiment primarily employs the five-point sampling method for sample collection on the 18th and 35th days of fermentation. During each sampling, 300–400 g of tobacco leaves are randomly collected from the middle, top-left, bottom-left, top-right and bottom-right of the tobacco stack, mixed and then placed into sterile centrifuge tubes. These tubes are stored in a −80°C freezer awaiting testing. Efforts are made to avoid collecting leaves from the surface and edges during sampling.

### 2.3 Determination of starch content in tobacco leaves

The starch content in tobacco leaves fermented for 18 and 35 days was determined using the Starch Content Assay Kit, purchased from Beijing Boxbio Science & Technology Co., Ltd. The soluble sugars and starch in the sample are separated using an eluent and the starch is then hydrolyzed by acid into glucose, which further reacts with anthrone to produce a blue-green furfural derivative. The absorbance of the product is measured at 620 nm to quantitatively determine the starch content. Each sample is analyzed in triplicate.

### 2.4 Metabolites and liquid chromatography-mass spectrometry (LC–MS) analysis

Among the analytical methods employed in metabolomics research, liquid chromatography-mass spectrometry technology occupies a significant proportion. Liquid chromatography (LC) possesses high resolution capabilities, enabling the efficient separation of complex mixtures, while mass spectrometry (MS) provides precise qualitative and quantitative information, overcoming the limitations of LC that rely on retention time or UV absorption. The liquid chromatography-mass spectrometry (LC–MS) technique can enhance the amount of information. Tobacco leaf samples fermented for 18 and 35 days, stored at −80°C, were sent to Majorbio for untargeted metabolomics analysis, with six replicates for each sample. The detailed parameters are as follows: Sheath gas flow rate: 50 Arb, Aux gas flow rate: 15 Arb, Capillary temperature: 320°C, Full ms resolution: 60,000, MS/MS resolution: 15,000, Collision energy: SNCE 20/30/40, Spray Voltage: 3.8 kV (positive) or 3.4 kV (negative).

### 2.5 Microbial DNA extraction from tobacco leaf surface and metagenomic analysis

Under sterile conditions, 20 g of tobacco leaf samples stored at −80°C were weighed and added to Erlenmeyer flasks containing 150 mL of sterile water, with three replicates for each sample. The flasks were shaken at 120 r/min in a constant temperature shaker for 30 min to disperse the microorganisms on the tobacco leaf surfaces. The supernatant in the flasks was filtered through 0.22 μm membrane filters. After filtration, the membranes were placed in sterile centrifuge tubes and sent to Majorbio for multiple displacement amplification (MDA) followed by metagenomic analysis.

### 2.6 Data analysis

The data on starch content in tobacco leaves, microbial diversity and microbial correlations were processed and analyzed using Excel 2019, SPSS 22.0, Origin 2024, Gephi0.10.1 and Cytoscape3.10.3 software. Metabolomics data and metagenomics data were handled at Majorbio Cloud^2^.

## 3 Results and analysis

### 3.1 Screening and identification of starch-degradation bacteria and their effects on the starch content of tobacco leaves

**Figure 1:**
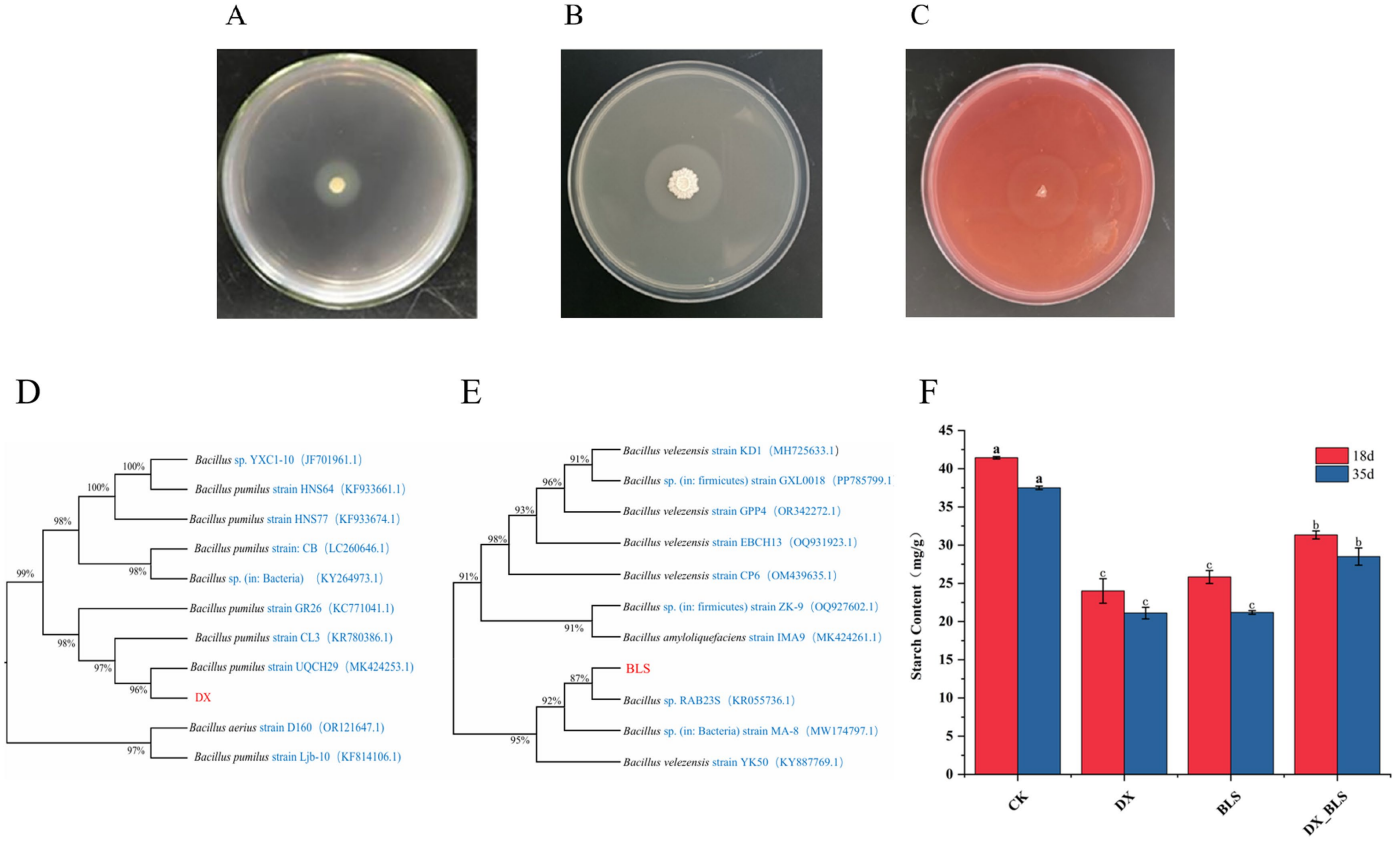
Screening, identification of starch-degradation bacteria and their starch degradation effects. Halo production of strain 1 and strain 2 on starch-containing medium **(A,B)**, halo production of strain 2 on sodium carboxymethyl cellulose medium **(C)**, phylogenetic trees of strain 1 and strain 2 **(D,E)**, starch content of cigar tobacco leaves **(F)**, where a, b and c represent significance.

This study employed the transparent circle method for the preliminary screening of amylase and cellulase activities in bacterial strains. By measuring the diameter of the transparent zones around the colonies, the ability of the strains to produce amylase was assessed, thereby screening for strains with the capability to degrade starch. In this experiment, strain DX and strain BLS, isolated from Dominican tobacco leaves at the end of fermentation, formed the largest transparent circles on starch-containing media, with D/d significantly higher than other strains, demonstrating strong starch-degradation capabilities, as shown in Figures 1A,B. Additionally, strain BLS formed a large transparent circle on sodium carboxymethyl cellulose media, indicating that strain BLS also possesses the ability to degrade cellulose (Figure 1C). Therefore, this experiment intends to use strain DX and strain BLS for subsequent fermentation tests.

The 16S rDNA gene sequences of strain DX and strain BLS were subjected to BLAST comparison on NCBI and reference sequences with high similarity were downloaded to construct their phylogenetic trees using MEGA11 software, as shown in Figures 1D,E. Strain DX clustered with *B. pumilus* in the GenBank database, leading to the preliminary identification of strain 1 as *B. pumilus*. Strain BLS clustered with *B. velezensis* in the GenBank database, resulting in the preliminary identification of strain BLS as *B. velezensis*.

This study systematically evaluated the dynamic impact of two strains of starch-degradation bacteria on the starch content of cigar tobacco leaves (Figure 1F). Experimental data revealed that the inoculation treatment significantly promoted the degradation of starch in the tobacco leaves (*p* < 0.01). After 18 days of fermentation, the starch content in DX, BLS and DX_BLS decreased to 24.00 ± 1.61 mg/g, 25.73 ± 0.73 mg/g and 31.34 ± 0.52 mg/g (*p* < 0.05), respectively, which was significantly lower than that of CK at 41.43 ± 0.16 mg/g (*p* < 0.05). After 35 days of fermentation, the starch content in DX, BLS and DX_BLS further decreased to 21.11 ± 0.76 mg/g, 21.19 ± 0.24 mg/g and 28.50 ± 1.13 mg/g (*p* < 0.05), respectively, which was significantly lower than that of CK at 37.49 ± 0.22 mg/g (*p* < 0.05). This indicates that both strains *B. pumilus* and *B. velezensis* (DX and BLS) possess strong starch-degradation capabilities. It is noteworthy that as the fermentation period extended to 35 days, the starch degradation effects in each treatment exhibited differentiated development, with BLS demonstrating the strongest sustained degradation capability, the degradation efficiency of the composite strain DX_BLS consistently remained lower than that of the single strain *B. pumilus* and *B. velezensis* (DX and BLS), which may be related to competitive inhibition among the strains.

### 3.2 Microbial diversity during the fermentation process of cigar tobacco leaves

**Figure 2:**
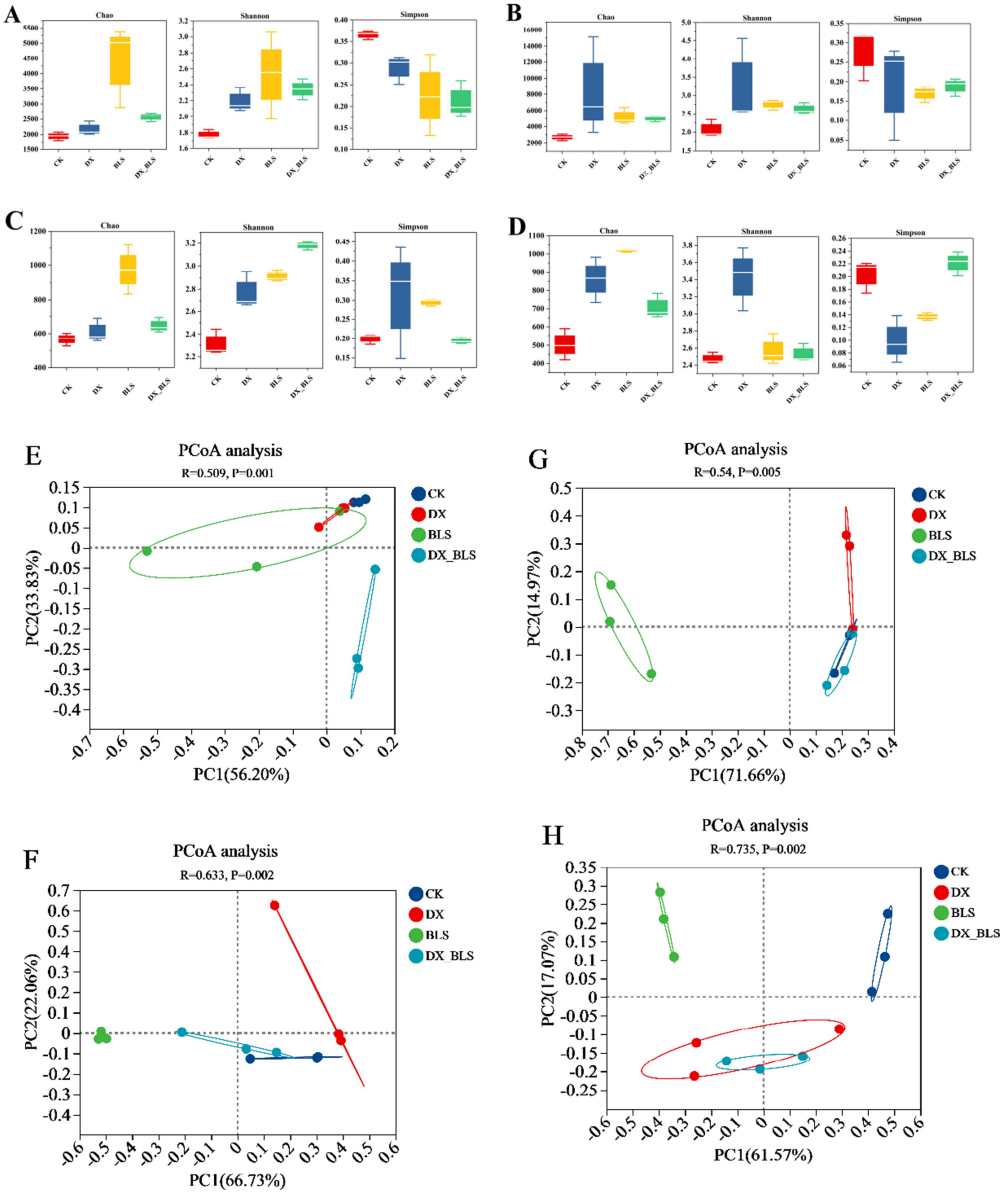
The differences in microbial diversity changes of tobacco leaves. Bacterial alpha diversity indices on the 18th and 35th days of fermentation **(A,C)**, fungal alpha diversity indices on the 18th and 35th days of fermentation **(B,D)**, bacterial beta diversity indices on the 18th and 35th days of fermentation **(E,F)**, fungal beta diversity indices on the 18th and 35th days of fermentation **(G,H)**.

This study employed a multi-dimensional indicator system to evaluate the dynamic changes in microbial α-diversity (Figures 2A–D and Appendix Table 1) and β-diversity (Figures 2E–H) during the fermentation of tobacco leaves.

After 18 days of tobacco leaf fermentation, the Chao index of bacterial communities (Figure 2A) indicated that CK had fewer species, while the bacterial community richness in DX, BLS and DX_BLS showed an increase compared to CK (*p* < 0.01). BLS had the highest number of species, significantly higher than CK, DX and DX_BLS. The analysis of Shannon and Simpson indices for bacterial communities (Figure 2A) revealed that CK had lower bacterial diversity, while DX, BLS and DX_BLS showed an increase in bacterial diversity compared to the CK, with Simpson indices decreasing to 0.29, 0.22 and 0.21, respectively. This indicates that the addition of amylolytic strains can enhance the diversity of bacterial communities. The trends in Chao, Shannon and Simpson indices for fungal communities (Figure 2C) were similar to those of bacteria, suggesting that the addition of starch-degradation bacteria during the first 18 days of cigar tobacco leaf fermentation can improve the richness and diversity of both bacterial and fungal communities.

After 35 days of tobacco leaf fermentation, the trends in the Chao, Shannon and Simpson indices (Figure 2B) of the bacterial communities were similar to those observed after 18 days of fermentation, indicating that starch-degradation bacteria continued to have a positive impact on the richness and diversity of the bacterial communities after 35 days of fermentation. The Chao index of the fungal communities (Figure 2D) showed that the community richness in DX, BLS and DX_BLS was higher than that in CK, suggesting that starch-degradation bacteria also positively influenced the changes in the richness of the fungal communities. The Shannon index and Simpson index indicated that DX was significantly higher than CK, suggesting that *B. pumilus* fermentation for 35 days could significantly enhance fungal community diversity. The Simpson indices of BLS and DX_BLS were close to those of CK, indicating that DX exhibited the strongest effect on enhancing fungal diversity during the later stages of fermentation.

Principal Coordinate Analysis (PcoA) was employed to compare the differences in microbial β-diversity among different treatments at 18 days and 35 days of fermentation (Figures 2E–H). An *R*-value close to 1 indicates significant differences between treatments. Therefore, the differences in bacterial communities (Figure 2F) and fungal communities (Figure 2H) among different treatments after 35 days of fermentation are greater than those in bacterial communities (Figure 2E) and fungal communities (Figure 2G) after 18 days of fermentation. This suggests that the fermentation time has a greater impact on inter-group differences than the different treatments. The clustering (*p* < 0.05) among CK, DX, BLS and DX_BLS at different fermentation stages indicates that there are differences in bacterial and fungal compositions among different treatments during the fermentation process.

### 3.3 Dynamic evolution of microbial communities during tobacco leaf fermentation

**Figure 3:**
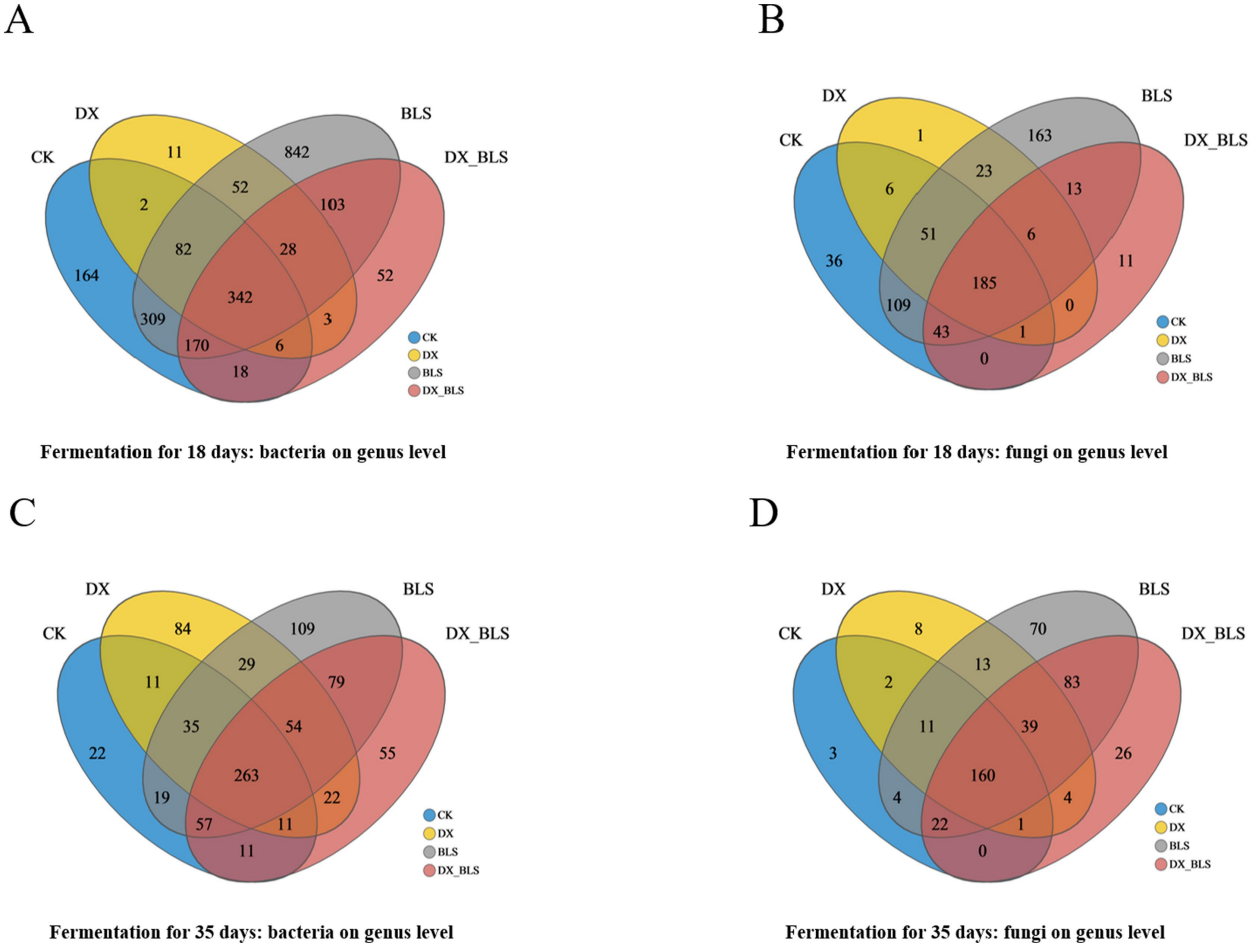
Dynamic evolution of microbial communities. Bacterial counts at the genus level for different treatment groups on the 18th and 35th days of fermentation **(A,C)**, fungal counts at the genus level for different treatment groups on the 18th and 35th days of fermentation **(B,D)**.

Venn diagrams illustrate the abundance of bacterial and fungal genera across four treatments at the genus level (Figures 3A–D). After 18 days of fermentation, BLS exhibited 842 unique bacterial genera (Figure 3A) and 163 unique fungal genera (Figure 3B), which were higher than those in CK, both DX and DX_BLS had fewer bacterial and fungal genera compared to CK. After 35 days of fermentation, DX, BLS and DX_BLS had 84, 109 and 55 unique bacterial genera, respectively, all higher than those in CK (Figure 3C), DX, BLS and DX_BLS had 8, 70 and 26 unique fungal genera, respectively, all higher than those in CK (Figure 3D). In summary, BLS exhibited the highest microbial diversity at both 18 and 35 days. The DX and DX_BLS showed an increase in the number of bacterial and fungal genera only in the later stages of fermentation and the number of unique fungal genera was generally lower than that of bacteria throughout the fermentation process.

### 3.4 The composition of microbial community structure and its impact on metabolic contributions during tobacco leaf fermentation

**Figure 4:**
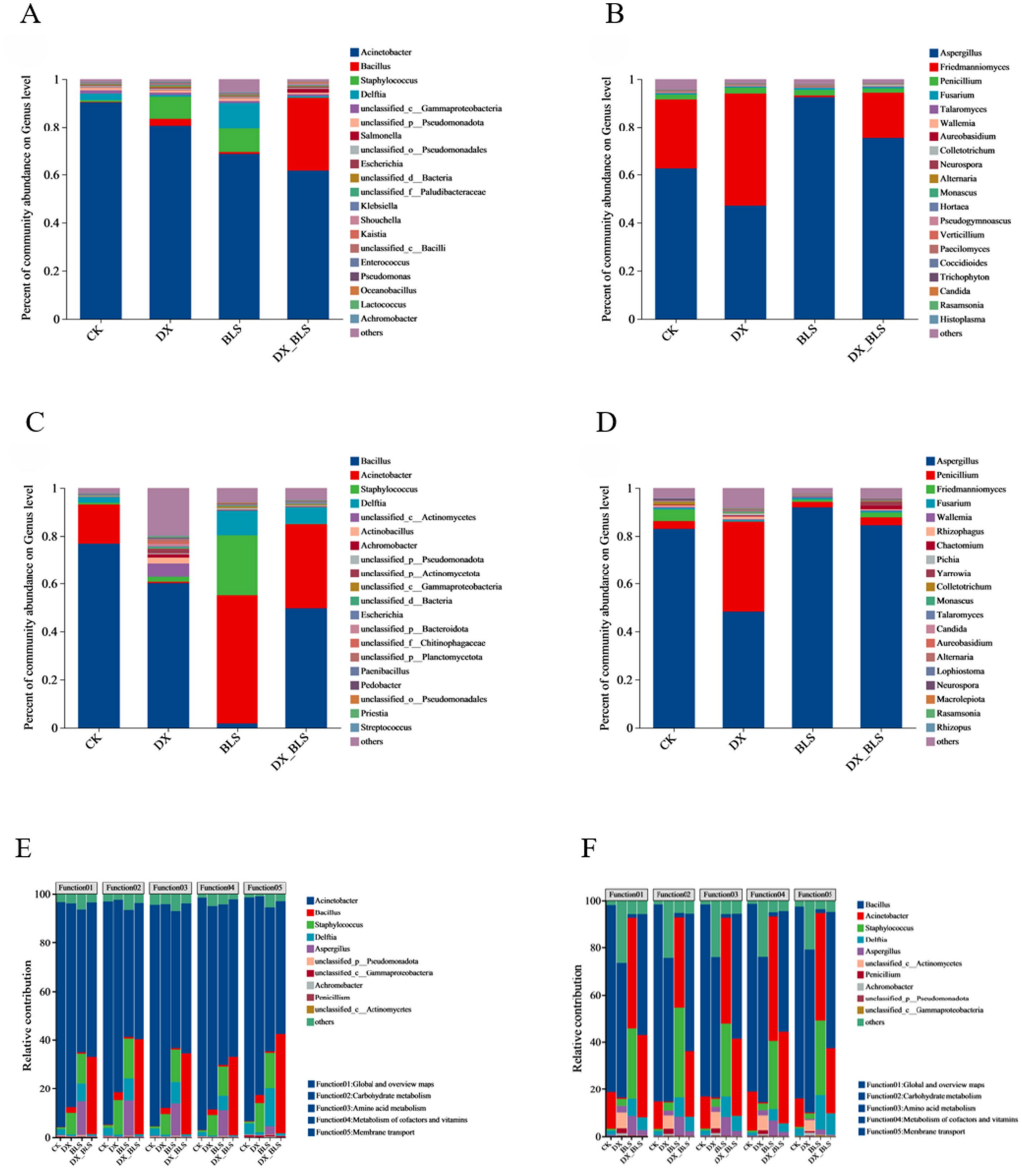
The composition of microbial communities and the influence of key bacterial communities on metabolic contribution rates. The top 20 dominant bacterial genera at the genus level on the 18th and 35th days of fermentation **(A,C)**, the top 20 dominant fungal genera at the genus level on the 18th and 35th days of fermentation **(B,D)**, association analysis between the top 10 dominant bacterial genera and the top 5 functions in terms of total abundance **(E)**, association analysis between the top 10 dominant fungal genera and the top 5 functions in terms of total abundance **(F)**.

To elucidate the differences in microbial community structure composition among different treatments at various fermentation stages, the top 20 dominant bacterial and fungal genera in terms of abundance were selected at the bacterial and fungal genus levels for tobacco leaf fermentation at 18 and 35 days. After 18 days of tobacco leaf fermentation, the dominant bacterial genera mainly include *Acinetobacter*, *Bacillus* and *Staphylococcus*. The relative abundance of *Acinetobacter* showed a significant decrease after the addition of starch-degradation bacteria. The relative abundance of *Bacillus* increased in DX, BLS and DX_BLS with the addition of starch-degradation bacteria, with the highest abundance observed in DX_BLS, significantly higher than the other three treatments, making it an important differential microorganism. *Staphylococcus* exhibited higher relative abundance in DX and BLS (Figure 4A). The dominant fungal genera mainly include *Aspergillus*, *Friedmanniomyces* and *Penicillium* (Figure 4C). After 35 days of tobacco leaf fermentation, the dominant bacterial genera remain *Bacillus*, *Acinetobacter* and *Staphylococcus*. Compared to CK, the relative abundance of *Bacillus* in DX, BLS and DX_BLS has significantly decreased. However, the relative abundance of *Acinetobacter* in BLS and DX_BLS is significantly higher than that in CK (Figure 4B). The dominant fungal genera mainly include *Aspergillus*, *Penicillium* and *Friedmanniomyces* (Figure 4D).

To analyze the functional contribution of specific species, an association analysis was conducted between species (the top 10 species in total abundance) and functions (the top 5 functions in total abundance) based on the correspondence between species and functions in the samples (Figure 4). In Figures 4E,F, the *x*-axis represents the sample names and the y-axis represents the metabolic contribution rate, indicating the contribution rate of different microbial genera to different functions. The dominant bacterial genera at 18 and 35 days of fermentation were *Acinetobacter*, *Bacillus* and *Staphylococcus* and the dominant fungal genera were *Aspergillus*, *Penicillium* and *Friedmanniomyces*. As can be seen from Figure 4E (Appendix Data Sheet 5) and Figure 4F (Appendix Data Sheet 6), the top 5 functions by total abundance include Function01: Global and overview maps, Function02: Carbohydrate metabolism, Function03: Amino acid metabolism, Function04: Metabolism of cofactors and vitamins and Function05: Membrane transport, with a particular focus on Function02: Carbohydrate metabolism. Through analyzing the metabolic contribution rates of dominant microbial genera to carbohydrates during the tobacco leaf fermentation for 18 days, it was found that the carbohydrate metabolic contribution rate of *Bacillus* in treatments with added starch-degradation bacteria DX, BLS and DX_BLS was significantly higher than that in the CK, *Staphylococcus* exhibited higher metabolic activity in DX and BLS, the contribution rate of *Aspergillus* significantly increased in BLS and DX_BLS, *Penicillium* showed a unique metabolic advantage in BLS. Through analyzing the metabolic contribution rates of dominant microbial genera to carbohydrates during the tobacco leaf fermentation for 35 days, it was found that the metabolic contribution of *Acinetobacter* significantly increased in BLS and DX_BLS, *Staphylococcus* maintained high metabolic activity in DX and BLS, the carbohydrate metabolic contribution rates of *Aspergillus* and *Penicillium* in DX, BLS and DX_BLS with added starch-degradation bacteria were significantly higher than those in CK. Therefore, *Bacillus*, *Acinetobacter*, *Aspergillus* and *Penicillium* are the key microbial communities for carbohydrate metabolism and the metabolic contribution of fungi significantly increased after 35 days of fermentation. The key bacterial community gradually shifted from *Bacillus* to *Acinetobacter*, indicating that the addition of starch-degradation bacteria enhanced the metabolic activity of the functional microbial community.

### 3.5 Analysis of differential metabolites in different treatments

**Figure 5:**
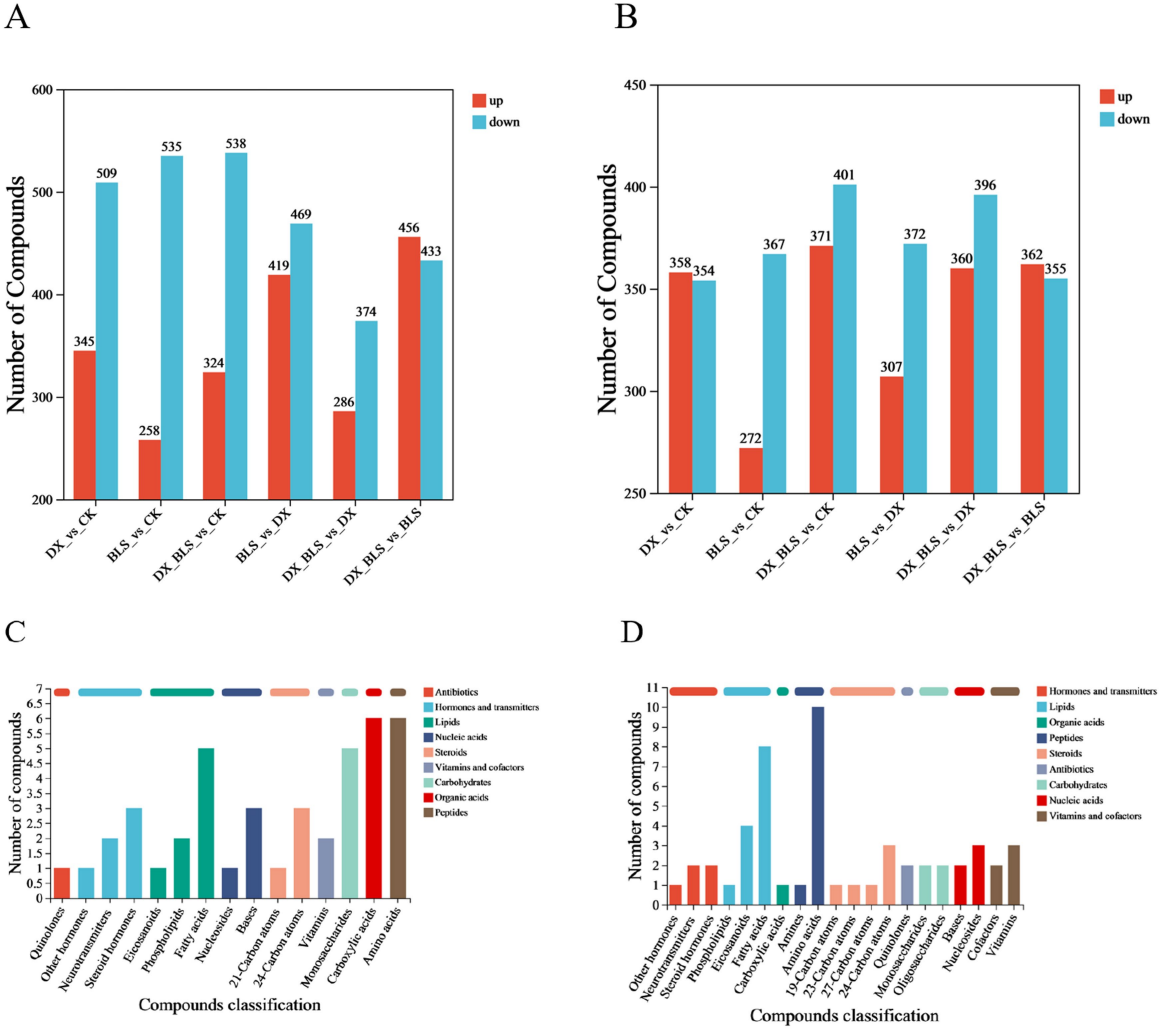
Comparative bar chart of differential metabolites among different treatment groups and statistical chart of compound classification. The bar charts of differential metabolites in different treatment groups after 18 and 35 days of fermentation, where red indicates upregulated differential metabolites and blue indicates downregulated differential metabolites **(A,B)**, statistical classification charts of differential metabolites after 18 and 35 days of fermentation **(C,D)**.

To investigate the impact of starch-degradation bacteria on metabolites during tobacco leaf fermentation, this study conducted pairwise comparisons of different treatment groups at 18 and 35 days of fermentation, screening for differential metabolites (*p* < 0.05, VIP > 1). Figures 5A,B displays bar charts of up-regulated and down-regulated metabolites in different treatment groups, with red representing up-regulated metabolites and blue representing down-regulated metabolites.

After 18 days of tobacco leaf fermentation (Figure 5A), a total of 854 differential metabolites were screened in the comparison between CK and DX, among which 345 were up-regulated and 509 were down-regulated, in the comparison between CK and BLS, a total of 793 differential metabolites were screened, among which 258 were up-regulated and 535 were down-regulated, in the comparison between CK and DX_BLS, a total of 862 differential metabolites were screened, among which 324 were up-regulated and 538 were down-regulated, in the comparison between DX and BLS, a total of 888 differential metabolites were screened, among which 419 were up-regulated and 469 were down-regulated, in the comparison between DX and DX_BLS, a total of 660 differential metabolites were screened, among which 286 were up-regulated and 374 were down-regulated, in the comparison between BLS and DX_BLS, a total of 889 differential metabolites were screened, among which 456 were up-regulated and 433 were down-regulated.

After 35 days of tobacco leaf fermentation (Figure 5B), a total of 712 differential metabolites were screened in the comparison between CK and DX, with 358 upregulated and 354 downregulated, 639 differential metabolites were screened in the comparison between CK and BLS, with 272 upregulated and 367 downregulated, 772 differential metabolites were screened in the comparison between CK and DX_BLS, with 371 upregulated and 401 downregulated, 679 differential metabolites were screened in the comparison between DX and BLS, with 307 upregulated and 372 downregulated, 756 differential metabolites were screened in the comparison between DX and DX_BLS, with 360 upregulated and 396 downregulated, 717 differential metabolites were screened in the comparison between BLS and DX_BLS, with 362 upregulated and 355 downregulated.

Classification and statistical analysis of differential metabolites across different treatment groups revealed that after 18 days of tobacco leaf fermentation, the differential metabolites were primarily fatty acids, monosaccharides, carboxylic acids and amino acids (Figure 5C). The changes in monosaccharides may suggest the effective breakdown of polysaccharides (such as cellulose or starch) by starch-degradation bacteria, releasing small molecular sugars like glucose. After 35 days of tobacco leaf fermentation, the differential metabolites were mainly fatty acids and amino acids (Figure 5D). The persistent differences in amino acids might reflect the slow degradation of proteins or other metabolic activities of microorganisms promoting the production of amino acids.

### 3.6 Analysis of the interaction relationships among microbial communities

**Figure 6:**
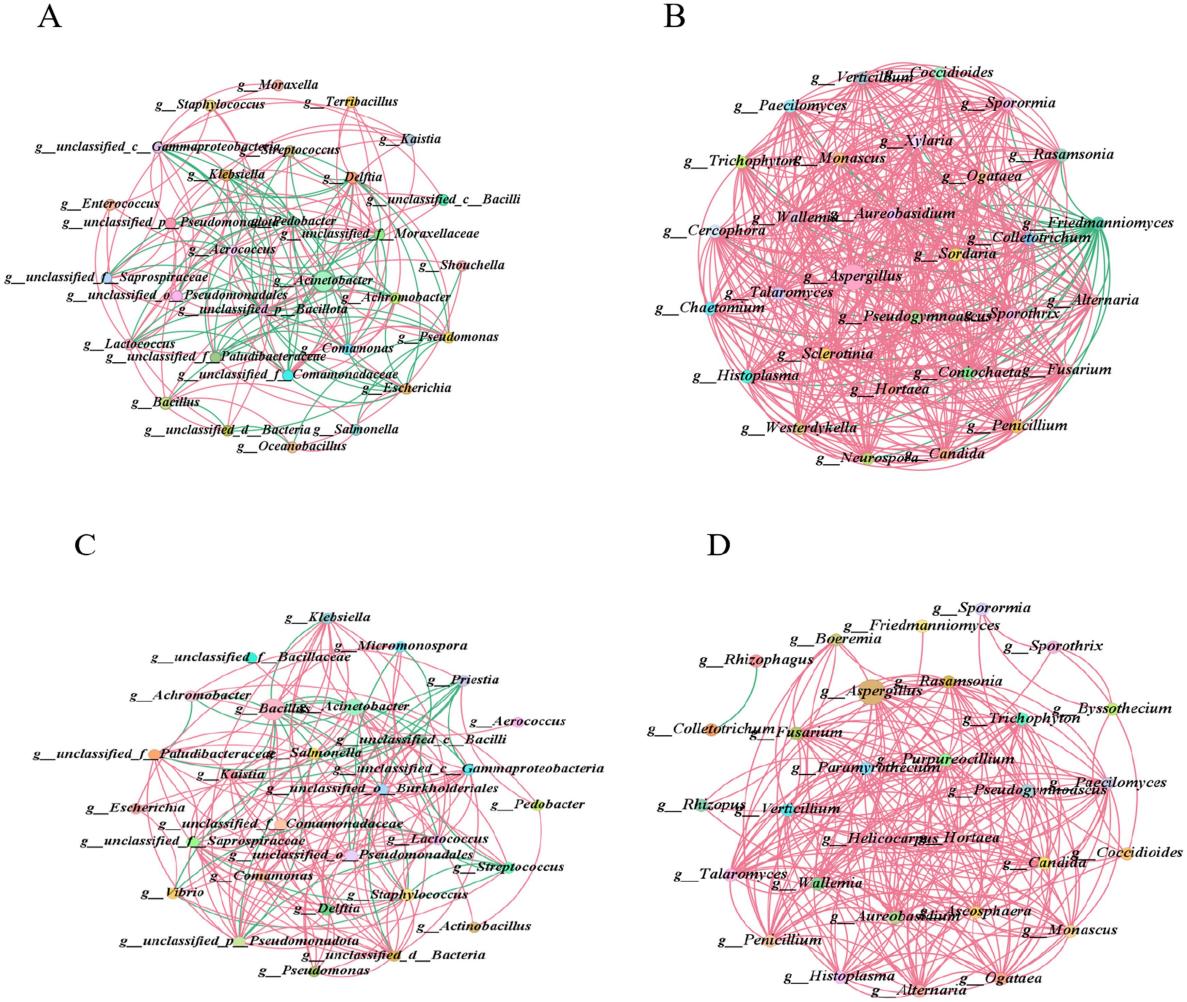
Microbial community interaction network. Interaction networks among the top 30 dominant bacterial genera in tobacco leaf fermentation on the 18th and 35th days **(A,C)**, interaction networks among the top 30 dominant fungal genera in tobacco leaf fermentation on the 18th and 35th days **(B,D)**. Red lines represent positive correlations, while green lines present negative correlations.

To elucidate the interaction relationships among microbial communities during the 18 days and 35 days fermentation processes of cigar tobacco leaves and to identify key species that significantly influence the structure and function of microbial communities, this study conducted correlation analysis using Pearson’s method (*p* < 0.05, |*r*| = 0.5). The top 30 dominant bacterial genera and fungal genera were selected for association analysis and the microbial interaction networks at the 18 days and 35 days fermentation stages were compared. The results are shown in Figure 6.

In the bacterial co-occurrence network diagram of tobacco leaf fermentation on day 18 (Figure 6A and Appendix Data Sheets 2, 4), positive correlations accounted for 56.29%, while negative correlations made up 43.71%. The bacterial genera with relatively high abundance were *Acinetobacter*, *Bacillus* and *Staphylococcus*. *Acinetobacter* interacted with 14 genera, showing positive correlations with 7 of them and negative correlations with the other 7 genera. *Bacillus* interacted with 7 genera, exhibiting positive correlations with 5 of them and negative correlations only with *g_unclassified_d__Bacteria* and *Pseudomonas*. *Staphylococcus* interacted with 4 genera, all of which were positive correlations. In the fungal symbiotic network diagram of tobacco leaf fermentation on day 18 (Figure 6B), positive correlations accounted for 93.53%, while negative correlations made up 6.47%. Among the fungal genera with relatively high abundance were *Aspergillus*, *Friedmanniomyces* and *Penicillium*. *Aspergillus* interacted with 29 genera, showing a negative correlation only with *Friedmanniomyces* and positive correlations with the other 28 genera. *Friedmanniomyces* interacted with 26 genera, all of which were negative correlations. *Penicillium* interacted with 26 genera, all of which were positive correlations.

In the bacterial co-occurrence network diagram of tobacco leaves after 35 days of fermentation (Figure 6C), positive correlations accounted for 80.39%, while negative correlations accounted for 19.61%. The bacterial genera with relatively high abundance were still *Bacillus*, *Acinetobacter* and *Staphylococcus*. *Bacillus* interacted with 14 genera, showing positive correlations with *Escherichia*, *Streptococcus*, *Micromonospora* and *Vibrio*, while the rest were negative correlations. *Acinetobacter* interacted with 12 genera, exhibiting a negative correlation only with *Achromobacter*, with the rest being positive correlations. *Staphylococcus* interacts with 14 genera, showing negative correlations with *Streptococcus*, *Micromonospora* and *Vibrio*, while exhibiting positive correlations with the remaining genera. In the fungal co-occurrence network of tobacco leaf fermentation at 35 days (Figure 6D), positive correlations account for 99.55% and negative correlations account for 0.45%. The fungal genera with relatively high abundance are *Aspergillus*, *Penicillium* and *Friedmanniomyces*, all of which display positive correlations with other fungal genera.

This indicates that in the microbial community symbiotic networks of cigar tobacco leaves during both fermentation stages, the bacterial genera with relatively high abundance are consistently *Bacillus*, *Acinetobacter* and *Staphylococcus*, while the fungal genera with relatively high abundance are consistently *Aspergillus*, *Penicillium* and *Friedmanniomyces*. Positive correlation is the predominant interaction between dominant microbial genera and other microbial genera. Positive correlation indicates that dominant microbial genera may coexist with other microbial genera in forms of symbiosis, cooperation, mutualism, or syntrophy, which helps support the stability of microbial community structure and jointly promotes certain metabolic activities.

### 3.7 Analysis of changes in starch metabolic enzymes during the fermentation process of cigar tobacco leaves

**Figure 7:**
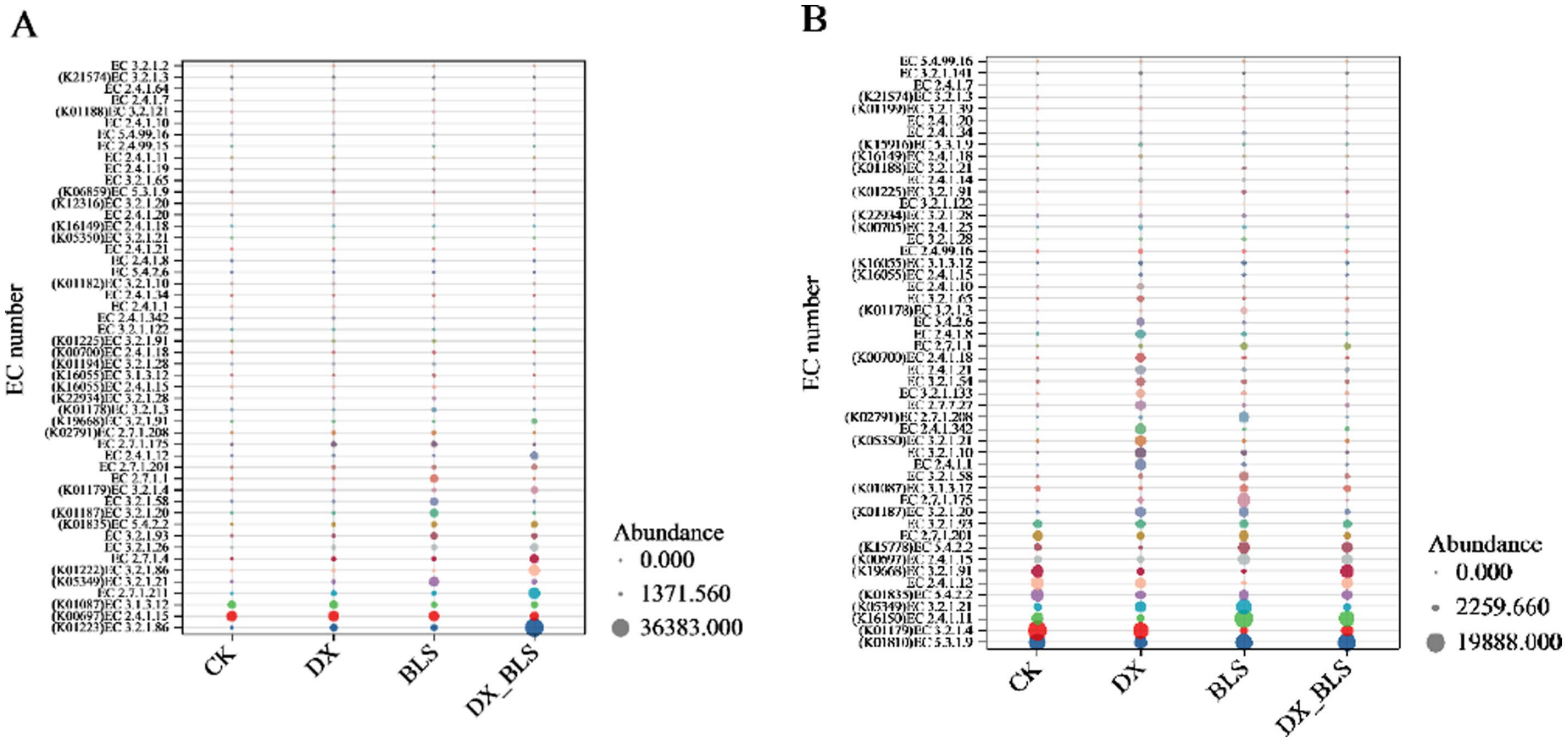
Bubble plot of abundance differences of starch metabolic enzymes among different treatments. The top 50 abundant starch metabolic enzymes screened after 18 days and 35 days of fermentation **(A,B)**.

During the fermentation process of cigar tobacco leaves, starch metabolic enzymes play a crucial role in the conversion of carbohydrates. Based on the KEGG database analysis, 71 types of starch metabolic enzymes were identified at 18 days of fermentation, which decreased to 69 types at 35 days of fermentation. The differences in the abundance of the top 50 enzymes among different treatments were compared using a bubble chart (Figure 7).

Tobacco leaves were fermented for 18 days (Figure 7A and Appendix Table 4), by comparing the abundance of enzymes with the same EC numbers across different treatments (represented by the size of the dots), the impact of adding starch-degradation bacteria on enzyme expression can be observed. As shown in Figure 7A, except for K00697 (EC 2.4.1.15) and K01087 (EC 3.1.3.12), which showed lower abundance in DX_BLS and BLS compared to CK, other key enzymes exhibited higher expression levels in the treatments with added starch-degradation bacteria (DX, BLS and DX_BLS). It is noteworthy that at 18 days of fermentation, the key starch metabolic enzymes alpha-amylase (EC 3.2.1.1) and glucoamylase (K12047, EC 3.2.1.3) were only detected in BLS and β-amylase (EC 3.2.1.2) had the highest relative abundance in BLS. This indicates that the addition of starch-degradation bacteria can significantly promote the production of starch metabolic enzymes in the early stages of cigar tobacco leaf fermentation and *B. velezensis* has the unique function of specifically inducing the synthesis of certain key starch metabolic enzymes.

Tobacco leaves were fermented for 35 days (Figure 7B and Appendix Table 5), compared to the 18 days fermentation, both the variety and abundance of starch metabolic enzymes exhibited a declining trend by the 35 days fermentation, which may be associated with the reduced consumption of starch-based substrates during the later stages of fermentation (Figure 7B). The key enzymes cellulase (K01179, EC 3.2.1.4), glycogen branching enzyme (EC 2.4.1.12) and glucan phosphorylase (EC 2.7.1.201) showed lower abundance in the treatments with added starch-degradation bacteria (DX, BLS, DX_BLS) compared to CK. This phenomenon suggests that the addition of starch-degradation bacteria may accelerate the starch metabolic process, leading to reduced expression levels of related enzymes during the later stages of fermentation. It is particularly noteworthy that β-amylase (EC 3.2.1.2) was not detected in CK at 35 days of fermentation, but was detected in all treatments with added starch-degradation bacteria (DX, BLS, DX_BLS), further confirming the sustained impact of starch-degradation bacteria on the starch metabolic pathway.

### 3.8 Enrichment analysis of differential metabolite metabolic pathways

**Figure 8:**
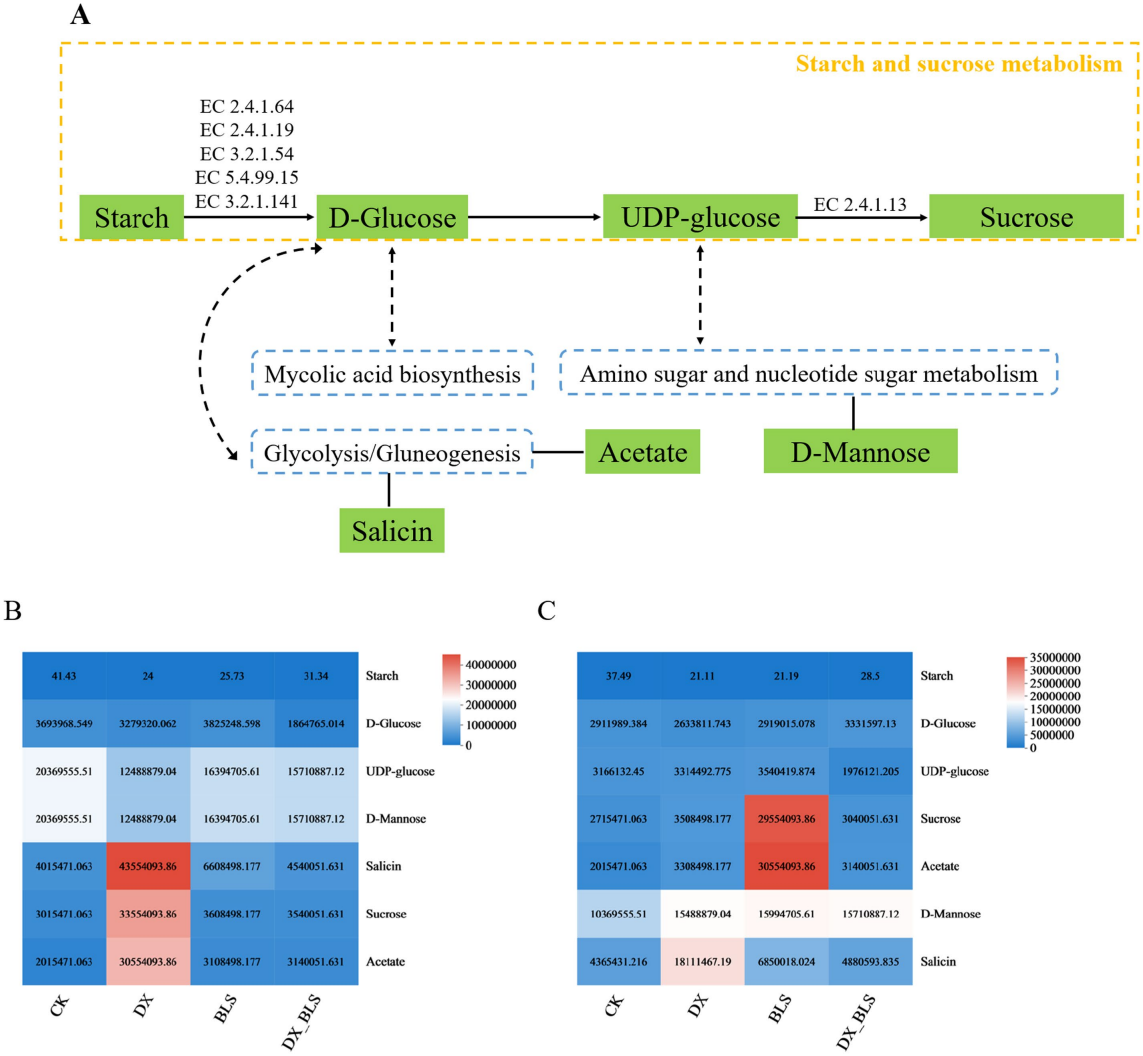
Starch metabolism-related pathways **(A)**. Heatmaps of the relative contents of related compounds in the starch metabolic pathway after 18 days and 35 days of fermentation **(B,C)**.

Through metabolic pathway enrichment analysis of differential metabolites in the four treatments, it was found that at 18 and 35 days of tobacco leaf fermentation, the differential metabolites were significantly enriched in four pathways related to starch metabolism: starch and sucrose metabolism and its subordinate pathways: amino sugar and nucleotide sugar metabolism, glycolysis/gluconeogenesis and mycolic acid biosynthesis (Figure 8A).

Starch and sucrose metabolism serves as the core pathway, where starch is progressively broken down into d-Glucose through the synergistic action of key enzymes EC2.4.1.64, EC2.4.1.19, EC3.2.1.54, EC5.4.99.15 and EC3.2.1.141. This process subsequently leads to the generation of UDP-glucose, which ultimately synthesizes sucrose under the catalysis of enzyme EC2.4.1.13. In this context, d-Glucose, serving as a metabolic hub, not only participates in the core pathway of starch metabolism but also connects its downstream pathways, including mycolic acid biosynthesis and glycolysis. Within the glycolysis/gluconeogenesis pathway, salicin and acetate are involved in the starch metabolism during the fermentation process of cigar tobacco leaves. Meanwhile, UDP-glucose, as another key hub molecule, participates in starch metabolism while bridging amino sugar and nucleotide sugar metabolism. In this pathway, d-Mannose is closely related to the starch metabolism during the fermentation process of cigar tobacco leaves.

After 18 days of tobacco leaf fermentation, the relative contents of starch metabolism-related compounds salicin, sucrose and acetate in the treatment groups (DX, BLS, DX_BLS) with added starch-degrading bacteria were higher than those in the CK group, with DX showing better results; however, the relative contents of d-Glucose, UDP-glucose and d-Mannose were lower than those in the CK group (Figure 8B). After 35 days of tobacco leaf fermentation, the relative contents of compounds related to starch metabolism—sucrose, acetate, d-Mannose and salicin—were higher in the treatment groups with added starch-degrading bacteria (DX, BLS, DX_BLS) compared to CK, with DX and BLS showing better effects. However, the relative content of d-Glucose in DX was lower than in CK and the relative contents of UDP-glucose and d-Mannose in DX_BLS were lower than in CK (Figure 8C). This might be due to the reaction system’s rate being too fast, leading to the accumulation of downstream products such as sucrose, salicin and acetate.

### 3.9 Correlation analysis of microbial communities and environmental factors

**Figure 9:**
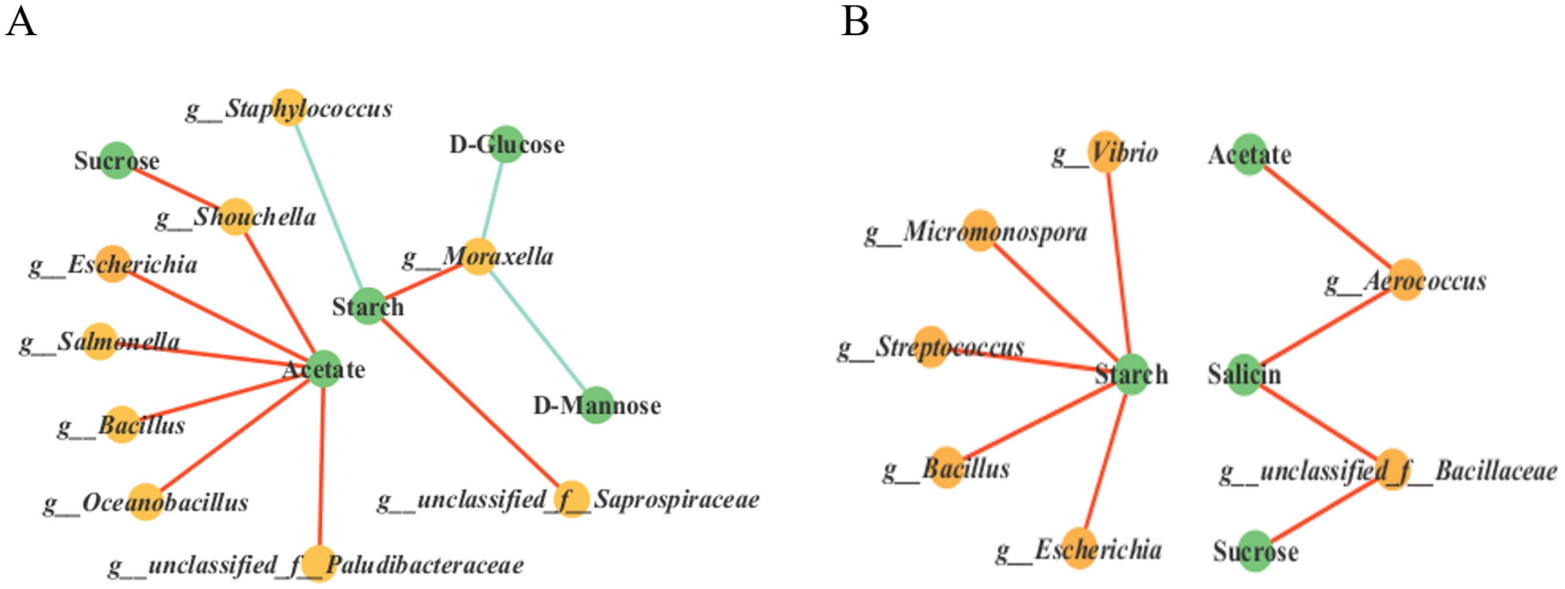
Multi-factor correlation network analysis. Correlation network diagrams of the top 30 dominant bacterial genera, starch content and starch metabolism-related compounds in tobacco leaves fermented for 18 days and 35 days **(A,B)**. The red lines represent positive correlations, while the green lines represent negative correlations.

To investigate the interaction between microbial communities (top 30 bacterial genera in abundance) and environmental factors (starch content, metabolic intermediate compounds) during the fermentation of cigar tobacco leaves, Pearson correlation network analysis was conducted (*p* < 0.05, |*r*| ≥ 0.5) and the results are shown in Figures 9A,B. The correlation network analysis results after 18 days of tobacco leaf fermentation (Figure 9A and Appendix Data Sheet 3) showed that acetate was significantly positively correlated with *Shouchella*, *Escherichia*, *Salmonella*, *Bacillus*, *Oceanobacillus* and *g_unclassified_f_Paludibacteraceae* indicating that these bacterial genera may promote acetate accumulation through metabolic activities. Sucrose was significantly positively correlated with *Shouchella*, suggesting that this bacterial genus may be directly involved in sucrose synthesis. Starch is significantly negatively correlated with *Staphylococcus*, suggesting that this genus may promote starch degradation by secreting amylase, starch is positively correlated with *g_unclassified_f_Saprospiraceae* and *Moraxella*, indicating that these genera may inhibit starch decomposition or utilize other carbon sources. d-Glucose and d-Mannose are significantly negatively correlated with *Moraxella*, suggesting that this genus may preferentially consume these monosaccharides. In summary, bacterial genera such as *Shouchella*, *Escherichia*, *Salmonella* and *Bacillus* promote starch metabolism by regulating the accumulation of acetate and sucrose, *Staphylococcus*, on the other hand, may directly participate in starch degradation.

The correlation network analysis results after 35 days of tobacco leaf fermentation (Figure 9B) showed that starch was significantly positively correlated with *Bacillus*, *Escherichia*, *Streptococcus*, *Micromonospora* and *Vibrio*, possibly reflecting the dependence of these genera on starch or its degradation products during the later stages of fermentation, acetate was significantly positively correlated with *Aerococcus*, indicating that acetate metabolism in the later stages of tobacco leaf fermentation might be driven by specific genera, sucrose and salicin were both significantly positively correlated with *g__unclassified_f__Bacillaceae*, suggesting that this genus might be involved in the metabolism of sucrose and salicin.

## 4 Discussion

### 4.1 Screening of starch-degradation bacteria and functional verification

In this study, the screened strains of *B. pumilus* and *B. velezensis* both demonstrated strong starch degradation capabilities, a finding consistent with existing research. This result aligns with the findings of Zhou et al. (2024), whose study showed that *P. amylolyticus* effectively reduces the starch content in the upper leaves of flue-cured tobacco. Their results indicated that the starch content in the leaves of the treatment group with the added strain was less than 40 mg/g, while the starch content in the treatment group without the added strain was greater than 40 mg/g. The results of this study show that the starch content in tobacco leaves from different treatment groups changed after 18 and 35 days of fermentation. The starch content in tobacco leaves treated solely with the starch-degrading bacterium *B. pumilus* was 24 ± 1.61 mg/g and 21.11 ± 0.76 mg/g, respectively, the starch content in tobacco leaves treated solely with the starch-degrading bacterium *B. velezensis* was 25.73 ± 0.73 mg/g and 21.19 ± 0.24 mg/g, respectively, the starch content in tobacco leaves treated with a combination of the starch-degrading bacteria *B. pumilus* and *B. velezensis* was 31.34 ± 0.52 mg/g and 28.50 ± 1.13 mg/g, respectively, the starch content in the control group was 41.43 ± 0.16 mg/g and 37.49 ± 0.22 mg/g, respectively. This indicates that the starch-degrading strains screened in this study possess strong starch-degrading capabilities.

### 4.2 The influence of starch-degradation bacteria on the diversity and structure of microbial communities

Through systematic analysis of bacterial and fungal populations at the genus level in different treatment groups fermented for 18 and 35 days, it was found that the number of unique fungal genera was generally lower than that of bacteria throughout the fermentation process. The lower number of unique fungal genera compared to bacteria may be attributed to the rapid growth rate of bacteria, which allows them to quickly occupy ecological niches, the complex cell wall structure of fungi leading to low DNA extraction efficiency and the insufficient coverage of fungal reference databases. Analysis of bacterial and fungal α-diversity and β-diversity revealed that the addition of starch-degrading bacteria significantly increased the community richness and diversity of both bacteria and fungi. This indicates that after the addition of starch-degrading bacteria, the microbial community in the fermentation environment may, through nutritional competition (Hu W. et al., 2022), secretion of metabolites and interactions, ultimately form a more functional fermentation environment while enhancing microbial diversity (Su et al., 2024; Zhang et al., 2023).

In terms of microbial community structure composition, the addition of starch-degrading bacteria significantly enriched several key functional microbial groups. In the bacterial community, key microbial genera such as *Acinetobacter*, *Bacillus* and *Staphylococcus* were notably enriched, while in the fungal community, key microbial genera such as *Aspergillus*, *Penicillium* and *Friedmanniomyces* were significantly enriched. These microorganisms are not only closely related to starch degradation but also influence the production of major volatile flavor compounds (VFCs) through enzymatic metabolism and the Maillard reaction (Wu X. et al., 2023). *Acinetobacter*, as a key functional bacterium in cigar tobacco fermentation, efficiently synthesizes various aldehyde and ketone flavor compounds such as benzaldehyde and phenylacetaldehyde, significantly enhancing the solanesol content and sensory qualities (fullness, sweetness, etc.) of tobacco leaves (Wang et al., 2024). Research has also found a mutualistic symbiotic relationship between *Acinetobacter* and *Bacillus*, jointly promoting the formation of flavor compounds (Zheng et al., 2022). *Bacillus* secretes multifunctional hydrolase systems including amylase, cellulase (Dai et al., 2020) and glycosidase (Haure et al., 2022), effectively degrading macromolecular substances such as starch, cellulose and carotenoids, thereby significantly improving the quality of tobacco leaves. Its metabolic activities not only reduce the irritancy of smoke (Liu et al., 2021), but also promote the formation of key aroma substances such as β-ionone and megastigmatrienone, thereby enhancing the sensory quality of tobacco (Zhang L.-Y. et al., 2024). *Staphylococcus* can extensively utilize various carbon and nitrogen sources, degrade proteins and carbohydrates and facilitate the synthesis of flavor compounds such as terpenes and phenylalanine derivatives (Hu et al., 2019; Mainar et al., 2016). The unique salt and heat tolerance characteristics of this bacterial genus enable it to function stably in fermentation environments, continuously transforming and generating volatile flavor components through amino acid and lipid metabolic pathways (Song et al., 2024). In this study, amino acids consistently emerged as the primary differential metabolites after 18 and 35 days of tobacco leaf fermentation. This phenomenon suggests that the addition of starch-degrading bacteria may have increased the accumulation of free amino acids by promoting protein degradation or microbial metabolism, thereby providing essential precursors for the synthesis of volatile flavor substances and ultimately optimizing the quality of the tobacco leaves. *Aspergillus* can efficiently degrade macromolecular substances such as starch, protein and cellulose (Wang et al., 2020) by secreting multifunctional enzyme systems including amylase, protease (Zhou et al., 2021) and cellulase (Florencio et al., 2016). Therefore, the significant enrichment of key functional microbial communities (such as *Acinetobacter*, *Bacillus*, *Staphylococcus*, *Aspergillus* and *Penicillium*) is closely related to starch degradation, flavor substance synthesis and the improvement of tobacco leaf quality (Li et al., 2020).

### 4.3 Starch degradation rate and analysis of key enzymes

In terms of starch content, this study found that inoculation with strains *B. pumilus* and *B. velezensis* (DX and BLS) significantly reduced the starch content in cigar tobacco leaves, validating the effectiveness of these two strains in starch degradation. Metagenomic analysis revealed that the types and quantities of enzymes related to starch metabolism exhibited dynamic changes during the fermentation process. At 18 days, 71 types of starch-metabolizing enzymes were detected, while at 35 days, 69 types were detected. This may be associated with nutrient depletion (substrate reduction leading to downregulation of enzyme expression), accumulation of metabolic waste (inhibiting the activity of some enzymes), decreased cellular activity and shifts in metabolic direction (Streb and Zeeman, 2012; Grennan, 2006). Among them, α-amylase (EC 3.2.1.1), β-amylase (EC 3.2.1.2) and glucoamylase (EC 3.2.1.3) are key enzymes for starch degradation (Antranikian, 1990; Chia et al., 2004; Niittylä et al., 2004; Fulton et al., 2008). These enzymes hydrolyze the α-1,4-glycosidic bonds, enabling the degradation of amylose to produce glucose, maltose, maltotriose and other oligosaccharides (Ryan et al., 2006; Awasthi et al., 2018). These enzymes collaboratively catalyze the conversion of starch into small molecular substances such as glucose and sucrose (Yamaguchi et al., 2013). In this study, after 18 days of tobacco leaf fermentation, monosaccharides became the primary differential metabolites, a phenomenon that may be closely related to the synergistic effects of starch-degradation enzymes.

### 4.4 Analysis of the KEGG metabolic pathway

KEGG pathway analysis revealed that starch degradation is primarily accomplished through four core metabolic pathways, with starch and sucrose metabolism serving as the dominant pathway. It works in concert with amino sugar and nucleotide sugar metabolism, glycolysis/gluconeogenesis and mycolic acid biosynthesis pathways.

This generation of metabolic networks may hydrolyze starch to produce small molecular substances such as UDP-Glucose, salicin, acetate and d-Mannose through key enzymes (EC2.4.1.64, EC2.4.1.19, EC3.2.1.54, EC5.4.99.15, EC3.2.1.141) (Yamaguchi et al., 2013), ultimately achieving the stepwise metabolism of starch and thereby improving the quality of tobacco leaves (Enez, 2021).

### 4.5 Research limitations and future directions

However, although starch-degradation bacteria may have a significant impact on the flavor compounds produced during the fermentation process, this study did not systematically evaluate the influence of starch-degradation bacteria on the flavor compounds of tobacco leaves, such as changes in key components like aldehydes, ketones and terpenes. Future research needs to combine sensory evaluations (softness, elasticity, combustion performance, etc.) with flavor compound analysis to more comprehensively elucidate the mechanisms by which strains enhance the quality of tobacco leaves, thereby optimizing the production process.

## 5 Conclusion

This study systematically elucidated the mechanisms of action of the starch-degradation bacteria *B. pumilus* and *B. velezensis* during the fermentation process of cigar tobacco leaves. Research findings indicate that the inoculation of starch-degradation bacteria significantly reduced the starch content in tobacco leaves. This is achieved through the secretion of α-amylase, β-amylase and glucoamylase, which synergistically catalyze the breakdown of starch into small-molecule sugars. The inoculation of starch-degradation bacteria also notably enhanced microbial diversity and enriched functional microbial communities associated with the synthesis of flavor compounds (such as *Bacillus*, *Acinetobacter*, *Staphylococcus*, and *Aspergillus*). As dominant microbial genera, *Bacillus* and *Aspergillus*, *respectively*, lead the initial degradation and complete hydrolysis of starch, forming a metabolically complementary network. Starch degradation is primarily accomplished through four core pathways: starch and sucrose metabolism (the dominant pathway), amino sugar and nucleotide sugar metabolism, glycolysis/gluconeogenesis and mycolic acid biosynthesis. Key intermediates (such as UDP-glucose) bridge multiple pathways for collaborative transformation, ultimately generating flavor precursor substances. This study confirms the potential of starch-degradation bacteria to optimize tobacco leaf quality by regulating microbial community structure and metabolic functions, providing a scientific basis for microbial regulation in cigar fermentation processes. Future research should combine flavor substance analysis with sensory evaluation to further validate the strains’ effectiveness in enhancing the overall quality of tobacco leaves.

## Data Availability

The datasets presented in this study can be found in online repositories. The names of the repository/repositories and accession number(s) can be found in the article/Supplementary material.

## Footnotes

^1^ http://www.ncbi.nlm.nih.gov

^2^ https://www.majorbio.com
